# Dress syndrome following carbamazepine exposure: A very early onset

**DOI:** 10.1192/j.eurpsy.2021.2047

**Published:** 2021-08-13

**Authors:** M. Kraiem, N. Charfi, E. Mhiri, I. Gassara, S. Omri, N. Smaoui, R. Feki, L. Zouari, J. Ben Thabet, M. Maalej Bouali, M. Maalej

**Affiliations:** 1 Psychiatry C, Hedi Chaker University Hospital, sfax, Tunisia; 2 Psychiatry C Department, Hedi chaker University hospital, sfax, Tunisia; 3 Hedi Chaker, psychiatry department, sfax, Tunisia

**Keywords:** Dress Syndrome, Carbamazepine, Early onset, eosinophilia

## Abstract

**Introduction:**

Drug reaction with eosinophilia and systemic symptoms (DRESS) is a severe cutaneous drug reaction characterised by both systemic and cutaneous clinical manifestation with a mean latency period of 3.9 weeks.

**Objectives:**

To underline the importance of an early diagnosis of DRESS SYNDROME.

**Methods:**

We reported a case of carbamazepine induced DRESS syndrome with atypical chronology of manifestations.

**Results:**

A 43-year-old man with no previous known medical history was admitted in psychiatry. He experienced a relapse of schizoaffective symptoms. In the last three years, the patient was treated by Valproic acid as a mood stabilizer. Because of the unavailability of this molecule, carbamazepine was prescribed in combination with antipsychotics. Three days later, the patient developed a high fever, hypotension, a pruritus, a facial oedema, a skin rash associated to lymphadenopathy. Laboratory findings showed a lymphopenia, eosinophilia and elevated liver chemistries. In order to define the case, RegiSCAR scoring system was used, and our case is categorized as probable with a score of five. Carbamazepine was discontinued upon clinical manifestationsand the patient was treated with systemic antihistaminic treatment associated to methylprednisolone with a good outcome. After 3 weeks, clinical and biological improvement were noted.

**Conclusions:**

Despite the absence of a delayed onset, typically between 3 weeks and 3months, we can diagnose dress syndrome 3 days after carbamazepine intake. This case highlights that psychiatrists should be aware of the risk of early onset dress syndrome associated with carbamazepine and they should monitor for warning symptoms from treatment initiation.

**Disclosure:**

No significant relationships.

